# The Effectiveness of International Non-Governmental Organizations’ Response Operations during Public Health Emergency: Lessons Learned from the 2014 Ebola Outbreak in Sierra Leone

**DOI:** 10.3390/ijerph15040650

**Published:** 2018-04-01

**Authors:** Yoon Ah Shin, Jungwon Yeo, Kyujin Jung

**Affiliations:** 1 Graduate School of Public and International Affairs, University of Pittsburgh, Pittsburgh, PA 15260, USA; yos28@pitt.edu; 2 School of Public Administration, University of Central Florida, Orlando, FL 32816, USA; Jungwon.Yeo@ucf.edu; 3 Department of Public Administration and Graduate School of Governance, Sungkyunkwan University, Seoul 02841, Korea

**Keywords:** international nongovernmental organization, public health emergency responses, Ebola Outbreak, Sierra Leone

## Abstract

International Nongovernmental Organizations (INGOs) have played critical roles in improving the quality of primary health care in ordinary time and, indeed, responding to epidemic crises in developing countries. Due to a lack of empirical research for effectiveness of their responding activities, the legitimacy and accountability of nonprofits’ engagement in the health crisis as a critical responder is doubted. This paper aims to examine the effectiveness of INGOs in a context of managing a fatal epidemic outbreak of Ebola in Sierra Leone during May–November, 2014; building healthcare infrastructures, providing medical supplies, educating local residents, and training response staffs. The analysis results show that development of healthcare infrastructures and provision of medical supplies have been significantly effective in terms of decreasing the severity of the crisis in chiefdoms. The findings imply that policy tools, which allow INGOs to enter to the field in a timely manner, can improve the effectiveness of INGOs’ responses in current and future epidemic outbreaks in developing countries where people suffer from a lack of health infrastructures.

## 1. Introduction

International Nongovernmental Organizations (INGOs) have extended their role in supporting public health around the world. Large-scale pandemics or epidemics, such as H1N1, Ebola, or Zika, have been major threats to human security and public health [1]. The growing frequency and impact of such large-scale diseases have often strained national governments’ response capacities. The complex problem situations often intervened by INGOs’ comprehensive and strategic operations in the field [1,2].

INGOs’ public health emergency operations have been imperative in developing countries [3,4]. In many developing countries, such as countries in Sub-Saharan Africa and Southeast Asia, INGOs usually work on the front line of public health response, establishing medical facilities, supporting local public health systems, educating locals, and providing shelters and necessities to victims [4]. In particular, INGOs have been widely acknowledged by their dedication and involvement in large-scale epidemic outbreaks, such as AIDS, yellow fever, malaria, and cholera, prevalent in many developing countries [4,5,6,7].

While INGOs have actively involved in managing immediate risks to human health and survival of victims in developing countries [6,8,9], the perspectives of how to evaluate their contributions and effectiveness of the activities those INGOs implement have conflicted. These conflicting perspectives come from using different definitions and purposes of individual INGOs to participate in emergency operations in response to public health crises [8,10]. In general, their effectiveness has been measured by logic models that provide detailed action plans [11]. The method gives more weights on implementing the planned actions than on understanding emerging needs and adapting operations to alleviate the situation in practice [3,4,5]. 

Yet, during public heath crises, situations do not develop orderly as planned. New problems and issues emerge as INGOs’ actions interact with contextual conditions. Multiple conditions may drive unpredictable and rapid situational changes. Thus, the current logic model approach may not precisely guide INGOs’ effective intervention in reducing the damages from public health crises [10].

This study aims to fill the gap in the current literature by empirically examining the effectiveness of INGOs’ public health emergency response operations in developing countries. This study answers the following question: “To what extends do INGOs’ response activities reduce damages in public health crisis in developing countries?” We infer the effectiveness of INGOs’ interventions by examining the impact of INGOs’ varying response activities on reducing Ebola outbreak in Sierra Leone in 2014. The findings contribute to INGOs’ response operations when managing public health threats in developing countries and devising strategies to enhance the effectiveness of existing response systems in practice. This study will support the streamlining and updating of INGOs’ response plans to maximize the benefit of early interventions and minimize the overall damage caused by similar epidemic outbreaks in the future.

This study is organized as follows. First, we provide an overview of INGOs’ intervention during public health crises. Second, the contextual background of this study, Sierra Leone, located in the West African region, is introduced. Third, we provide a review of studies that measure the effectiveness of health emergency responses and hypothesize the influence of INGOs’ interventions in managing the overall epidemic situation. The Section 4 introduces the utilized methods, and the results are presented in the subsequent section. Lastly, based on the findings, we suggest several policy strategies that can contribute to the performance of INGOs’ response operations in public health emergencies.

## 2. Ebola Outbreak in Sierra Leone in 2014

The first Ebola virus disease (EVD) case was confirmed in Sierra Leone in May 2014, and, even though it initially seemed possible to effectively respond to the outbreak in the first two months, by the end of December 2014 the number of confirmed cases and deaths had soared, totaling 11,751 confirmed cases and 3691 deaths [12]. A key principle of international humanitarian crisis response emphasizes the major role of national governments in coordination and strengthening of the local response capacity. Sierra Leone has very vulnerable national health systems that lack sufficient resources. The World Health Organization (WHO, Geneva, Switzerland) recommends a minimum of one healthcare worker for every 439 individuals. However, Sierra Leone had 5319 people for one health worker [13]. Furthermore, the national government spent very little money on essential health services. While the WHO suggested $86 per person, the Sierra Leone government spent only $14 [14,15]. Therefore, increasing healthcare facilities and medical resources, including medical staff, was a priority.

Since the EVD is transmitted through bodily contact, isolating infected people is the most urgent strategy to prevent further contagion. Experts attempted to completely isolate the infected people from society. Therefore, they thoroughly blocked contact with the outside world to prevent any possibility of EVD exposure. In the early stages of the EVD outbreak, several health organizations participated in increasing the number of beds in the public healthcare facilities in the affected countries and later also constructed EVD treatment centers (ETCs). In such situations, even local nonprofits with no experience of implementing health programs in the past participated in building a health infrastructure to increase the number of isolation units [16].

The major goal of public health education was to stop eating bushmeat, because bushmeat, including wild bats and monkeys, was suspected of acting as a bridge between animals and human beings and thus spreading the virus. The Sierra Leone government also announced similar bans on the sale of bushmeat, spreading alarm and dismay among consumers and the many people who made a living from the trade [17]. Furthermore, they had spread strong warning messages to encourage people to report any EVD symptoms in their families, emphasizing that “Ebola kills”, “Ebola is incurable”, and “There is no vaccine” [18]. Other important measures included banning traditional burial practices. Traditional burial practices were a major route to transmitting the EVD because local people wash the dead bodies and kiss them before burials. Therefore, public education was aimed at banning such traditional burial practices.

At the beginning of the response period, while the Sierra Leone government mainly focused on the international response, they rarely focused on local engagements such as training for local politicians, leaders, and volunteers [19]. Furthermore, because of the over-emphasis on medical provisions, the government could not take care of several extra factors. For example, affected mothers could not leave their families without implementing any measures for taking care of their family members, particularly children. This situation resulted in infected mothers trying to hide themselves, thus spreading EVD to the entire family. Even though some local nonprofits provided child care services, the service provision level was marginal. To deal with all the unexpected situations, response activities were more diversified than any other health crisis. A comprehensive examination of the effectiveness of diverse response activities would therefore contribute to the development of strategies to minimize damage at the early stage of any future public health crisis.

## 3. INGOs’ Response Effectiveness in Public Health Emergencies

In response to extreme events threatening a global health security, multi-scale and cross-sector response and relief operations are critical [20,21]. Particularly, such a scale-free response is necessary in developing countries where national governments do not have enough capacity to prevent further spread due to a lack of social, financial, and human resources. Multiple INGOs have been engaged in responding to pandemic and epidemic disasters in many developing countries. INGOs have often formulated plans and strategies and incorporated their resources and experiences, which are often unavailable to the public sector of developing nations [22,23].

INGOs’ public health response operations have different emphasis on an ultimate goal of health operations. Some INGOs focus on providing primary health care delivery [6]. In developing countries like Sierra Leone, marginal supplies of even basic health services cause huge mortality as a result of chronic but easily preventable and curable disease with a simple treatment, such as diarrhea, malaria, and yellow fever. When providing primary health care, INGOs focus on direct treatments for patients to decrease mortality by providing medical experts and appropriate health infrastructures. INGOs such as Doctors Without Borders (MSF, Geneva, Switzerland) focus on (1) building or expanding health care infrastructure and (2) providing medical supplies to patients during a public health crisis. These kinds of medical responses serve a critical role in not only mitigating the situation but also preventing the further spread of the disease.

Another focus of health operations is to increase community resilience by creating sustainable socio-economic environment, of which health is a part [24]. By engaging and empowering community members in the field of health operations, INGOs activities aim at encouraging these community members to adopt protective behaviors for themselves and other community members. Ultimately, the community members will serve to implement the activities they participated in with the INGOs. The community empowering activities include (3) health education to local residents and (4) training and consultancy to local health staff and leaders to support localized responses. In an epidemic situation, the community empowering activities also support (5) normal care activities, such as sanitation and provision of clean water and food, which may leverage their main public health response activities during the disaster [2]. The following are detailed discussions regarding public health response activities of INGOs.

### 3.1. Healthcare Infrastructure

According to a WHO response guide, the priority activity when responding to an epidemic situation is to establish isolation units for the infected to avoid contact with other people [16,25]. Rapid expansion of medical facilities to augment the surge capacity is critical for preventing the further spread of an infectious virus, particularly in a developing country suffering from a great shortage of health infrastructure. Most heath care centers in Sierra Leone do not have proper sewage systems due to a lack of water. Without constructed walls, treatment rooms or spaces are separated through temporary curtains or partitions. EVD, which spreads through bodily fluid, requires special types of treatment centers equipped with insulated sewage systems and completely separate rooms for patients according to their affected phases from a suspicious stage to a recovery stage [26]. Particularly, these specialized treatment centers hold suspicious or affected cases in quarantine rooms with proper treatment supports until those cases are completely free from the Ebola virus. As a result of providing appropriate Ebola treatment centers, INGOs are able not only to treat more Ebola patients but also contribute to preventing following spread of the virus by isolating the patients who are mobile virus carriers from their family and community members [27]. Therefore, increasing proper healthcare infrastructure may decrease the number of Ebola affected cases.

**Hypothesis** **1.** Increase in response activity to expand healthcare infrastructure will decrease the damage caused by an epidemic situation.

### 3.2. Medical Supplies

INGOs who participate in health service-delivery operations also provide medical supplies to affected communities. Along with an increase in facility-based surge capacity, providing medical supplies should follow supporting health facilities’ proper operations to increase the mitigation effectiveness of crisis situations [28]. The response of a developing country to an epidemic situation substantially depends on international donations due to a lack of domestic medical stocks. Ebola treatment centers need to be well equipped with medical resources, such as medicines, extra beds, and safety kits for patients and health workers, to treat patients while protecting health workers. INGOs deliver medical supplies from foreign donors into affected communities. The immediate delivery of medical supplies may facilitate timely patient admissions into the Ebola treatment facilities. The WHO points out importance of prompt delivery of medical supplies; a rapid provision of hospital beds decreased the number of newly affected people during the Ebola crisis [29]. Therefore, INGOs’ active participation in delivering medical supplies to affected communities may quicken the time to treat and isolate individual EVD-affected cases from communities, and hence decrease the total number of the Ebola affected cases.

**Hypothesis** **2.** Increase in response activity to provide medical supplies will decrease the damage caused by an epidemic situation.

### 3.3. Public Health Education

INGOs’ health education operations also serve a critical preventive function for further damage in the community. Education programs, including campaigns and advocacy, focus on providing information about risk, as well as specific guidelines for prevention and early detection of risk. These programs help local residents to understand risk factors and preventive actions. Such efforts raise communities’ awareness of the diseases and guide actions regarding prevention and treatment strategies [30]. For example, in Sierra Leone, traditional burial practice functions as very important rituals to show one’s sincerity and honor to a dead person. During the ritual, families and friends are in frequent contact with the corpse for several days [16]. However, such cultural practices can cause fast spread of the virus in a community. Therefore, INGOs’ health education programs targeting dissemination of disease information may help affected community by encouraging people to change dangerous behaviors [31].

**Hypothesis** **3.** Increase in response activity to provide public health education will decrease the damage caused by an epidemic situation.

### 3.4. Response Training

The success of early interventions depends on the skills and knowledge of the first responders in affected communities [32]. First responders need basic training, including first aid and public hygiene for the population at risk, as well as appropriate level of contextual knowledge to communicate with local people to facilitate response operations. In recognition of first responders, INGOs have provided response training to community leaders and public health staff, as first responders of their own communities, helping them make informed decisions at the ground level, as well as facilitating adaptive implementation of international approaches in local context. Response training enables those staff to detect and respond to a crisis in the first place from early on. Record indicates that Ebola cases reports made by community leaders and public health staff resulted in more relief items and medical support in the affected communities. In addition, well-trained community leaders may translate the international response approaches and contextualize them to fit into the local environment. Especially, such response training of community leaders is important in a tribe-based society like Sierra Leone, where the leaders have strong authority that has an influence on community members’ day to day decisions and behavior. Therefore, we hypothesize INGOs’ community-based response training may decrease the number of infected people.

**Hypothesis** **4.** Increase in response activity to provide response training will decrease the damage caused by an epidemic situation.

### 3.5. Direct Care of the Vulnerable Population

INGOs provide direct services to the vulnerable, thus building community resilience [33]. The first goal of direct care is to ensure that people maintain a fundamental level of nutrition during a crisis. A crisis situation may affect the nutritional status of the population and increase their susceptibility to the given situation [34]. Especially, INGOs’ nutrition care services are beneficial to affected communities when most work places and markets are closed due to government regulation aiming to minimize human contacts by limiting people’s movement [13]. Second, direct service includes isolations of suspicious cases to prevent further disease spread. In particular, INGOs’ participation in isolating those suspicious cases plays a critical role in highly affected rural communities lacking Ebola treatment centers and medical supplies. This service also provides interim care programs for children who are separated from their infected or suspicious parents. Therefore, this service helps those infected or suspicious parents to willingly be separated to limit any potential damages not only for their own families but also for communities. Those children sent to children-interim centers get psychological supports to avoid trauma side-effects possibly caused by long-term separation from their parents. Lastly, in the field, INGOs continue tracking the statuses of affected parents—their location and recovery status—to ensure a family reunion of the affected or suspicious parents with their children after recovery. As these direct care services help to isolate infected cases, they will subsequently help limit further spread of the disease within a household, ultimately decreasing the possibility of the disease spreading in a community. Therefore, INGOs’ active participation in direct care activities will decrease the number of infected people.

**Hypothesis** **5.** Increase in response activity to provide direct care services decreases the damage caused by an epidemic situation.

### 3.6. Existing Local Health Capacity

People in developing countries have suffered from chronic health problems, such as infant mortality, malnutrition, and poor maternal health, and from communicable diseases, such as diarrhea and tuberculosis (TB). These chronic health problems in developing countries are caused by a limited access to health care due to a short of resources. As insufficient resources are combined with inadequate access to necessary drugs and treatments, the general health condition has deteriorated [35]. Healthcare services also serve health education and health monitoring for a population, along with medical treatments, ultimately promoting health awareness and preventing possible disease. To improve the quality of the health condition in a community in developing countries, residents should, most importantly, be physically accessible to the healthcare services. Therefore, we include the number of health facilities—district hospitals (DH) and peripheral health units (PHUs)—in each community to indicate the condition of local health infrastructure as control variables, assuming that a community with more healthcare facilities is less vulnerable to an infectious virus.

**Figure 1:**
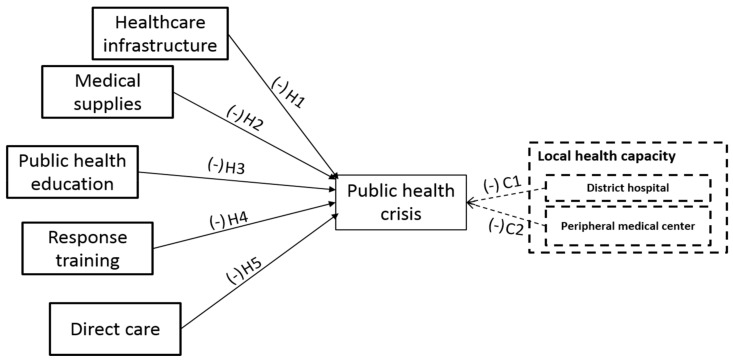
INGOs’ Response Effectiveness Framework.

Figure 1, below, depicts how we hypothesize the INGOs’ response effectiveness during public health emergencies. 

## 4. Research Design and Methods

### 4.1. Data Collection

To examine the hypotheses, we focused on response operations during the first six months (May–November 2014) at the smallest administrative level at which INGOs implemented response activities: the chiefdom. We collected data from 7 March to 25 March from diverse archives—MapAction, GeoNode, and the Ministry of Health and Sanitation of Sierra Leone. The data from MapAction (2014) [36] provides the number of EVD infected people in each chiefdom in Sierra Leone from 31 March to 6 November, 2014. The data from Ebola GeoNode (2004) [37], which is a partnership platform with the United Nations Office for the Coordination of Humanitarian Affairs for sharing geospatial data, analysis, and maps related to the Ebola emergency response, provides the population of each chiefdom and local healthcare facilities, including DHs and PHUs. The Ministry of Health and Sanitation of Sierra Leone provides information of participating (number of) INGOs, ranging from international to local-based nonprofits, which were published by UNICEF (MHSSL, 2014) [38]. This information shows the lists of participating INGOs and their specific activities in each chiefdom. To understand INGOs’ response activities during the Ebola outbreak, we exchanged correspondence with a program officer to obtain details regarding the Ebola response operations in the field [39].

### 4.2. Variables

To examine the effectiveness of INGO’s response operation, we measured the impact of INGOs’ response activities on the damages caused by EVD epidemics. We operationalize the dependent variable—damages caused by Ebola—as the severity of the Ebola situation. The dependent variable is measured as a ratio of the confirmed cases—the number of confirmed Ebola cases divided by population size—in each 149 chiefdoms. Ebola spreads through human contact; the larger population in a community, the higher chances to be affected. Even if the mere number of Ebola-affected cases is higher but in the higher population, the situation could be less severe than other communities with smaller affected cases inside smaller populations. Thus, we weighted community population with the number of affected cases to measure the genuine impact of Ebola epidemic crisis in a community.

Counting the number of INGOs that participate in a particular activity in each chiefdom indicates the extent of operation of each activity. INGOs’ activities are categorized into healthcare infrastructure, medical supplies, education, response training, and direct care provision. Healthcare infrastructures aim at constructing new healthcare facilities with original management and network systems so as to increase the capacity to accept a surge of affected people. Medical supplies include activities to supply beds, medical supplies, and protective gowns for medical staff. Education aims at promoting the public awareness by helping people to understand the disease and relevant preventive behaviors. INGOs participating in educational supports deliver educational pamphlets, educational posters, community campaigns, radio discussions, and public rallies. Response training involves training community health workers, peer educators, and community stake-holders/key decision makers, including chiefs, religious leaders, councilors, women’s leaders, youth leaders, traditional leaders, contact tracers, and surveillance officers. Multiple INGOs participated in direct care services during the EVD outbreak. As many children lost their parents or were left alone when their parents were affected, the INGOs provided childcare interims, family tracing and reunification services as well as food supplies.

To control the impact of the existing medical infrastructure in each chiefdom, we also count the number of DHs and PHUs, such as community health clinics, community health posts, and maternal and child health posts.

### 4.3. Statistical Model

A regression model assumes that the outcome variable is numeric and normally distributed, or binary. However, in clinical research, the outcome variable cannot meet the normal distribution condition. Often, it is a count of rare events such as the number of EVD infection cases occurring in a population over a certain period. Such instances of research should include regression analysis by modeling the dependent variable Y as the estimate of the outcome using some or all the explanatory variables.

In the case of binary regression, the fact that probability lies between 0 and 1 imposes a constraint. The normality assumption of multiple linear regression is lost, along with the assumption of constant variance. Without these assumptions, the *F* and *t* tests have no basis. The solution is to use the logistic transformation of probability p or logit p, such that log_e_(p/1 − p) = β0 + β1Χ1 + β2Χ2 … βnΧn. The β coefficients could now be interpreted as increasing or decreasing the log odds of an event, and expβ (the odds multiplier) could be used as the odds ratio for a unit increase or decrease in the explanatory variable.

In addition, we adopted our model to deal with the over-dispersed count data. Table 1 shows a big gap between the number of maximum and minimum affected cases in communities. Poisson regression may be applicable in modeling count data. The regression model operates in a condition that conditional variance is the same as the conditional mean. However, a dispersion test [40] of our data shows that the Ebola-affected cases are overly dispersed (29.36844, *p*-value = 4.875 × 10^−5^), indicating that the number of affected cases is not normally distributed across the country. In other words, while some communities have a minimal number of affected cases, other communities have a multitude of affected cases. Therefore, as a generalized model of Poisson regression, a negative binomial statistics model is adopted to analyze the over- dispersed count data [40] by using statistic program R. Since we use the incident ratio—the number of Ebola confirmed cases divided by community population size—our interpretation of the data analysis result will provide the event probability influenced by the independent variables we set.

$${\mathrm{Log}}_{e}\;\left(\mathrm{The}\;\mathrm{number}\;\mathrm{of}\;\mathrm{confirmed}\;\mathrm{Ebola}\;\mathrm{cases}/\mathrm{community}\;\mathrm{population}\right)\;=\;\;\;\beta 0+\beta 1\mathrm{HI}+\beta 2\mathrm{MS}+\beta 3\mathrm{EDU}+\beta 4\mathrm{RT}+\beta 5\mathrm{DC}+\beta 6\mathrm{DH}\;+\beta 7\mathrm{PHU}+e$$

## 5. Analysis Results

The results from the negative binomial analysis in Table 2 indicate that all variables are significant at 0.001 or 0.001 significance levels. However, the direction and impact size vary.

Of these, the most effective function of INGOs in reducing the severity of the crisis situation is healthcare infrastructure development (β = −1.315 at the 0.001 level). When one more INGO participates in healthcare infrastructure development in a chiefdom, the probability of confirmed cases decreases by 73%. Activities for health capacity development require preparations for an unexpected situation in which a crowd of patients has to be received. Along with increasing the number of healthcare facilities, they plan ways to coordinate with other medical facilities to handle an overflow of patients and efficiently manage health workers. As EVD becomes very fatal after a very short incubation period; isolating patients is very important to prevent its further spread. By enlarging the medical capacity, patients have an increased chance of getting proper treatment in isolation units. This result confirms H1 and supports the importance of infrastructure building in the early response.

Providing medical supplies is also effective for decreasing the probability of confirmed cases (β=−0.585). As we hypothesized (H2), a unit increase in medical supplies decreases the probability of confirmed cases by 44%. As Sierra Leone has a very fragile medical infrastructure, people can obtain neither preventive medical treatment before the infection nor sufficient medical cares with strong medicines and aggressive treatments from medical facilities after the infection. INGOs’ provision of medical goods both to the medical facilities and to individual households is critical for meeting medical needs. By getting preventive medical treatments, people could be less vulnerable to EVD. 

The provision of response training to community leaders and health staff effectively works to decrease the severity of the crisis situation (β=−0.752); when response training activities increase by one unit, the probability of confirmed cases decreases by 53%. This confirms H4. Response training activities focus on key people directly related to the Ebola response activities, such as health workers, peer educators, and decision makers (leadership), rather than the general public. During the outbreak, many health workers died from infection. Health workers have a greater chance of not only being exposed to EVD patients but also exposing themselves to other health workers and other key responders for surveillance. Educating and training decision makers in chiefdoms is also critical. In Sierra Leone, the public supports unofficial leadership, such as that by religious chiefs and the elders in a chiefdom, more than the government. In other words, people rely on the statements and opinions of local leaders in the chiefdom. Therefore, it is more effective for educated and trained community leaders to propagate preventive and responsive actions to the local residents especially when those recommended preventive and responsive actions are against their cultural conventions and norms.

Unlike our expectations, other functions by INGOs such as health public education programs (H3: β=0.115) and direct care service provisions (H5: β=0.495) are positively associated with the probability of confirmed cases. However, we can interpret the results based on the contextual conditions. The effects of the public health education and direct care functions may not be spontaneously evident. The members of a community may not be serious about education, especially during the early response period we analyze. Their cultural convention and daily survival could take priority over official and scientific knowledge. Therefore, they may purposely resist accepting new knowledge. The contextual conditions could also impede the effectiveness of direct care services that attempt to reduce the probability of confirmed cases. In society, suffering from infection may be regarded as a spiritual curse or social stigma. Locals do not trust special treatments from foreigners. Therefore, regardless of the readiness of direct care services, many affected people try to hide themselves or are hidden by their family members. Moreover, isolation of the patients for prevention purposes could increase resistance to reporting suspected symptoms and infections by raising fear of separation from their family rather than fear of EVD. For these contextual reasons, both education and direct care services are positively associated with confirmed cases.

Lastly, the results of control variables are interesting. While the existence of district hospitals (DHs) does not influence to decrease the probability of Ebola affected cases, peripheral medical centers (PHUs) rather increase the probability of the confirmed cases (β=0.088). In other words, the existence of PHUs increases the probability of the confirmed cases by 109%. We can find this interesting context from a 2014 survey that shows that a significant level of distrust exists in national health services [41]. Of the respondents, 40% reported that they paid a bribe for healthcare, while 68% reported that they experienced a shortage of basic medical care. Moreover, more than 50% experienced a lack of medicine/supplies, long waiting times, absent doctors, expensive services, and lack of attention or respect [41]. Thus, such negative perceptions of medical facilities may discourage people from making early reports of their Ebola-suspicious or -affected cases. This delay in reporting could increase the probability of confirming subsequent cases.

## 6. Discussions

Disaster management is a continuous feedback process ultimately aimed at developing long-term community resilience. Resilience is defined as the “capacity for collective action in the face of unexpected extreme events that shatter infrastructure and disrupt normal operating conditions” ([42], p. 33). To improve the resilience of health functions in a community, five aspects of the health operation should be particularly strengthened [43]. First, community residents should have feasible access to health services, such as public health, healthcare, and social services. Second, community residents should be highly efficacious in dealing with unexpected challenges during major disruptions or disasters through proper information and education. Third, wide ranging communication and collaboration is required among the public, INGOs, and the community. Fourth, at-risk community residents need to be substantially engaged as major actors in disaster management to build and strengthen community health resilience. Fifth, empowered residents play an important role in helping one another during a crisis. Building a strong social connection among residents is essential during ordinary times.

INGOs played a critical role in coping with EVD epidemic crises by covering those five activities. As major health treatment providers, INGOs established proper healthcare centers with medical supplies to reach out to a larger number of vulnerable populations. We identified that these efforts significantly reduced the probability of confirmed cases even during the early response period. With respect to the significance, we argue that medical infrastructures with adequate level of medical supplies could have further reduced the affected cases in the short term, whereas community engagement activities such as public education, response training, and direct care activities could have more effective in controlling the epidemic cases in a long term. The community engagement activities aimed at people’s behavioral changes driven by increasing awareness would also serve as an important means to prevent other epidemic outbreaks in the future.

The response training for local leaders was a good example of improving the devastation, since it may encourage incorporation of local context in the response operation. Training and empowering local leaders enables them to play the role of first responders in communicating with and helping local residents. Communities in Sierra Leone had been ruled by paramount chiefs and several ruling families until recently [44]. In local, reputation-based society, engaging local leadership may lead to successful response operations during a crisis but also to building strong community capacity to mitigate and prepare for a potential epidemic risk. The findings of this research also support the fact that INGOs’ response training to local leaders and health staff has a higher impact than providing medical supplies with regard to the decrease in probability of confirmed cases. This result shows the significance in involving local leaders in response operations to help them make informed decisions and disseminate preventive guidelines to their communities during disasters and crises.

Through the public health education program, such as campaigns and advocacy programs, INGOs might intend to influence residents’ awareness and raise efficacy when coping with EVD crisis. However, the contradictory results in this research indicate that the social reputation or authority of educators of such programs would matter for a successful implementation. Especially, in a tribal community, local residents may accept new information from the sources that they trust, such as their community leaders, rather than an education program itself. Thus, we argue that health education as a critical part of risk communication should be provided in ordinary times as well. The implementation of new information and knowledge takes time. Through multiple trials and errors, people would lean and adjust their conventional behaviors and norms. If people are already aware of how epidemic diseases spread through public health education in advance to disasters, the education would be more effective during the early response period of epidemic crises.

## 7. Conclusions

These findings present practical implications highlighting the role of INGOs in building effective interagency communication and collaboration in response to epidemics. INGOs should be closely connected to timely risk information, which is mainly issued and also transmitted by principal government agencies. The findings also provide the key notion that INGOs’ pivotal role in bridging between government agencies and residents enables not only to help them access risk information but also to facilitate proactive and response actions to mitigate further transmission.

To improve the current response systems, response activities during the crisis should continue after the crisis so that those changes, especially the training for community leaders and the public education programs, can strengthen a community mitigation and preventive plan. However, most INGOs cease their activities when the outbreak is terminated due to a lack of resources. The new established healthcare centers by INGOs are abandoned without a continuous management of the facilities and medical supplies. While governments obtain financial support from international organizations or other countries through multilateral agreements, local nonprofits substantially depend on philanthropy foundations and the private sector. While small-sized local nonprofits have developed local knowledge and mutual trust with residents for a long time, they have fewer chances than INGOs to obtain funding from philanthropy foundations and the private sector. As described above, responders’ understanding of local context including culture, norms, and values is critical in developing collective efforts from residents. Local nonprofits that have already established tight relationships with local residents may effectively provide services through new established healthcare centers while providing public health education. INGOs may reinforce these local nonprofits with technical and financial assistance. International organizations may consider ways to develop and sustain relationships with local nonprofits for a long-term disaster response operation in developing countries.

This research contributes to filling the gap in the current research by empirically examining the effectiveness of INGOs’ crisis response operations. The findings support INGOs’ legitimacy to work in a public health response operation. Furthermore, the assessment of INGO effectiveness can assist donors to invest in the right response operations by correcting their heuristic perception and improving their understanding about how to measure the effectiveness of INGOs’ operations in response to epidemic outbreaks. Thus, lax management of resources due to over- or under-funding could be resolved. By providing pre-conditions to increase the effectiveness of the response activities, this research will serve as a guideline to assist in developing appropriate designs of policy implementations, which ultimately supports pertinent allocation of relief resources.

We also identify several limitations of this study. First, the results of this research are heavily interdependent of the unique socio-economic-cultural contexts of affected countries and types of diseases. Thus, the findings may not be generalizable in developed countries due to the varying degrees of INGOs’ engagement public health response activities in those nations. Second, this research examined the effectiveness of each response activity only for a short-term early response period. The impact of each response could vary across early, peak, and late response periods. Therefore, we suggest future study to conduct meta-analyses that compare the effectiveness of INGOs’ response activities in different contexts and different response periods for different types of diseases.

## Figures and Tables

**Table 1:** Descriptive Analysis.

		Mean	S.D.	Min	Max
Dependent variable	Number of affected cases	17.31	54.11	0	423
	Population	24,071.36	16,317.45	2607	87,627
Independent variables	Healthcare Infrastructure	0.40	0.67	0	2
	Medical Supplies	0.44	0.63	0	3
	Education	13.26	6.88	3	33
	Response Training	0.69	0.88	0	4
	Direct Care	1.53	1.19	0	5
Control variables	District Hospitals	0.17	0.54	0	4
	Peripheral Medical Centers	6.35	4.93	0	34

**Table 2:** Analysis Results.

Variables		Estimate	Std. Error	exp (Estimate)
Independent Variables	(Intercept)	−9.847 ***	0.36	0.00
	Healthcare Infrastructure	−1.315 **	0.29	0.27
	Medical Supplies (MS)	−0.585 ***	0.28	0.56
	Education	0.115 ***	0.04	1.12
	Response Training	−0.752 ***	0.28	0.47
	Direct Care	0.495 ***	0.13	1.64
Control Variables	District Hospitals (DH)	0.141	0.29	1.15
	Peripheral Medical Centers (PMC)	0.088 ***	0.03	1.09

*** *p* < 0.001/** *p* < 0.01.
